# Anatomical and Surgical Implications of the Usage of Bichat Fat Pad in Oroantral Communication, Maxillary, Palatal, and Related Surgeries—Narrative Review

**DOI:** 10.3390/jcm12154909

**Published:** 2023-07-26

**Authors:** Kamil Nelke, Alicja Morawska, Bartłomiej Błaszczyk, Maciej Janeczek, Edyta Pasicka, Marceli Łukaszewski, Krzysztof Żak, Maciej Dobrzyński

**Affiliations:** 1 Maxillo-Facial Surgery Ward, EMC Hospital, Pilczycka 144, 54-144 Wrocław, Poland; 2 Academy of Applied Sciences, Health Department, Academy of Silesius in Wałbrzych, Zamkowa 4, 58-300 Wałbrzych, Poland; kzak@ans.edu.pl; 3 Department of Pediatric Dentistry and Preclinical Dentistry, Wrocław Medical University, Krakowska 26, 50-425 Wrocław, Poland; alicja.m.morawska@gmail.com (A.M.); maciej.dobrzynski@umw.edu.pl (M.D.); 4 Student Scientific Circle of Experimental Dentistry and Biomaterial Research, Faculty of Dentistry, Wroclaw Medical University, Bujwida 44, 50-345 Wrocław, Poland; 5 Department of Biostructure and Animal Physiology, Wrocław University of Environmental and Life Sciences, Kożuchowska 1, 51-631 Wrocław, Poland; maciej.janeczek@upwr.edu.pl (M.J.); edyta.pasicka@upwr.edu.pl (E.P.); 6 Department of Anaesthesiology and Intensive Care, Sokołowski Hospital, Sokołowskiego 4, 58-309 Wałbrzych, Poland; marceliluk@gmail.com

**Keywords:** Bichat fat pad, buccal fat pad graft, oroantral communication, palatal fistula, sinus closure

## Abstract

The buccal fat pad, also called the Bichat’s fat pad (BFP), is an encapsulated fat mass located in the cheek. This type of specialized fat mass can be used both as a pedicular or free graft in various surgeries and approaches. Due to its easy access from the oral cavity approach, it is commonly used for oroantral and palatal fistula closure. The knowledge of its anatomy and surrounding tissues plays a role in its mobilization and suturing onto the desired defect in the palatal or maxillary region. The BFP is mostly associated with the primary approach used for a fistula or bone surgery. Alternatively, the procedure can be performed with a single approach incision, which does not compromise the appearance or the function of the operating or adjacent areas. The most important inclusion criteria for BFP usage and surgical limitations are highlighted. The BFP is used for multiple purposes in reconstructive and oncology surgery and also has its use in esthetic and facial contouring procedures. The amount, volume, and shape of the BFP are mostly associated with the scope of their usage. The aim of the following narrative review is to present the surgical and anatomical implications of fat pads in maxillary and palatal surgeries.

## 1. Introduction

The Bichat fat pad (BFP), known as Bichat Ball, Bichat’s protuberance, or buccal fat pad, is a specialized mass of adipose tissue located in the buccal region [1]. This eponym derives from the name of French anatomist Marie-Francois Xavier Bichat (1771–1802), who described it for the first time at the beginning of the XIX century. The following anatomists conducted several experimental studies, which built the foundation for modern physiology and other branches of medicine [2]. The first suggestion for using the BFP appeared in 1977. Egyedi proposed the usage of the buccal fat pad for the closure of oro-antral and oro-nasal communications for the first time. In four presented cases, the author found that mobilization and shifting of the buccal fat pad combined with a free skin graft may be a simple and reliable method of obtaining closure of medium-sized defects [3]. The use of the BFP as a free graft was described by Neder in 1983 and as a pedicule graft by Tideman three years later. Mentioned scholars observed that the epithelialization of the graft occurs for 2 or 3 weeks without the necessity to cover this with skin grafts [4,5,6]. The BFP can be used in various surgical situations. Their usage can be divided into several aspects. Authors’ proposals include the following: functional (for example, covering of adjacent defects, scar revision surgery, fibrosis treatment, jaw bone necrosis, temporomandibular join ankylosis-related surgeries, and teeth root coverage), esthetic (for example, facial oval contouring and buccal esthetic procedures), and reconstructive purposes (oro-antral fistulas, bone defects coverage, oro-nasal fistulas coverage, cleft-related surgeries usage, palatal/maxillary defects after tumor treatment, peri-implant and peri-implantitis treatment, etc.) [6,7,8,9,10,11,12]. The majority of indications for BFP usage are related to its very good positioning in the facial region, good anatomical relations with the maxillary bone, hard and soft palate, easy dissection technique for harvesting and mobilization of this pedicled fat, along with good healing potential and adequate epithelialization with a low rate of complications when used [2,3,4,5,6,7,8].

The study authors performed a rigorous search in the following databases: PubMed, Scopus, Web of Science, Research Gate, and Google Scholar. From a total of 351 articles, only 78 meet the following terms for inclusion in this review: “oroantral fistula”, “oro-nasal-fistula”, “Bichat fat pad surgery”, “palatal defects”, “maxillary defects”, “bone defects”, “Bichat defect coverage”, “Bichat clinical and surgical relevance”, and “fat pad in oral cavity”. The following results were divided into detailed anatomy, surgical relevance, indications and contraindications, oroantral fistulas, maxillary wall defects, palatal defects, and other usage and complications subsections. Articles focusing on topics not related to the BFP’s surgical relevance and its surgical functional and reconstructive usage in the oral cavity were excluded.

The following narrative review is focused on the surgical and anatomical implications of fat pads in maxillary and palatal surgeries.

## 2. Detailed Anatomy

The buccal fat pad is distinct from subcutaneous fat. Its capsulated form is connected with various anatomical structures by ligaments and has four anatomical extensions. Because of this, and its location in the facial area, it has a good blood supply. It is a mass of specialized fatty tissue, the volume of which varies throughout life, estimated at 10 cm^2^, and has three independent lobes: anterior, posterior, and intermediate [1]. One of its special features includes some wrapping of a fibrous capsule that prevents it from being metabolized. The buccal fat pad is situated between the buccinator muscle medially, the anterior margin of the masseter muscle, and the mandibular ramus and zygomatic arch laterally [3]. It is attached by six ligaments to the maxilla, posterior zygoma, inner and outer rims of the infraorbital fissure, temporalis tendon, and buccinator membrane [4,5,6]. The following attachments and anatomical surroundings make the BFP a good material for surgeries not only in the oral cavity (maxilla and palatal bone defects) but also in the nasal cavity, orbital socket, temporo-mandibular joint area, and soft palatal and oropharynx area, as well as a good covering material for various bone defects in the area [1,2,3,4,5]. Its extensions towards the pterygoid, pterygopalatine, temporal, and deep maxillary area should be considered while planning its usage [3,4,5,6,7,8].

The anatomy of the buccal fat pad consists of the main body along with its extensions and processes. Researchers distinguish its six extensions, such as masseteric, superficial and deep temporal, pterygomandibular, sphenopalatine, and inferior orbital [7].

The BFP is divided into three lobes anatomically, each encapsulated by an independent membrane, fixed by membranous adhesions, and nourished by different blood supply sources. The body is located behind the zygomatic arch. The anterior lobe is triangular in shape and located below the zygoma. It extends to the front of the buccinator muscle, maxillary bone, and the deep space of the quadrate muscle of the upper lip and posterior to the zygomaticus major muscle. The intermediate lobe is situated in and around the posterior lobe, lateral maxilla, and anterior lobe. It is a membrane-like structure with thin fatty tissue in adults but is a prominent mass in children [4,5,6]. The posterior lobe lies in the masticatory and neighboring spaces. It extends up to the inferior orbital fissure and surrounds the temporalis muscle, and extends down to the superior rim of the mandibular body and back to the anterior rim of the temporalis tendon and ramus. It forms the buccal, pterygopalatine, temporal processes, and, according to some authors, a pterygoid process. Processes extend from the body into surrounding spaces such as the pterygomandibular and infratemporal fossa [3,5,7,8,9].

Special anatomical considerations in BFP surgery should include the position of the parotid gland salivary duct anteriorly and the buccal branches of the facial nerve laterally, while the buccal and transverse facial artery and vein might cross the pad in its superior aspect. The more lateral the dissection is performed, the closer to the SMAS layer (Superficial musculoaponeurotic system) and the possibility of damaging the zygomatic branches of the facial nerve. Presented anatomical facts might impact later complication rates [1,2,5,6,7,8,9,10,11,12,13].

The blood supply arises from the subcapsular vascular plexus originating from anastomosis of the facial, transverse facial, and internal maxillary arteries. The rich capillary network is derived from three branches of the maxillary artery: the deep temporal, buccal, and superior posterior alveolar arteries. Each lobe has a separate vascularization. Venous drainage is provided by the facial vein [10,11].

The anatomical surroundings of the BFP are quite interesting. It is located in very close proximity to other anatomical structures, like the oral cavity (maxillary tuberosity and pterygoid plates) and maxillary sinus, muscles (masseter, buccinator, and lateral and medial pterygoid), and salivary ducts, sometimes even accompanied by the accessory parotid gland. The anterior facial vein passes through the anterior–inferior margin of the lobe. The infraorbital vessels and nerves close the fat tissue complex. The parotid duct, known as Stensen’s duct, passes through the posterior part of the anterior lobe. The branches of the facial nerve lie on the outer surface of its capsule. Histological observations showed that autonomic ganglia-like structures, composed of nervous cells, can be found in the buccal aspect of this fat structure [7,8,9,11,12]. An interesting fact is that this portion of fat in the BFP, despite weight loss and exercise, does not reduce its size, shape, and volume. Because of the following anatomical features, it can also be a source of adipose stem cells [10,11,12,13,14,15]. These two facts, along with the pedicular nature of the BFP, good blood supply, and stable volume, might be related to good surgical results because of its usage in various oral cavity and adjacent areas surgeries [5,7,10,11,12,15,16,17].

The volume, shape, and size of the BFP can influence the shape of one’s facial contour. Racz et al. postulated that the BFP performs three main functions. First, it amortizes and creates a slipping platform for the working masticatory muscles, filling the masseter-zygomaticus-buccinator space. In infants, it resists the negative pressure which acts in the buccal cavity during sucking. Moreover, its rich venous net, provided with valve-like structures, may be implicated in the exo-endocranial blood flow by means of the pterygoid plexus [12].

## 3. Surgical Relevance

Reconstructions within the zygomatic-maxillary area cause many problems for the maxillofacial surgeon, both esthetic and functional reasons. The anatomical structure of the buccal fat pad and its close relation to the zygomatic-maxillary region are of particular surgical relevance. The buccal extension of the BFP is the only process that can be easily separated from its adjacent tissues. Its location, consistency, and size make it a suitable choice for the reconstruction of maxillary, palatal, and adjacent bone defects [9]. The buccal extension of the posterior lobe is located the most superficially below the parotid duct. It is also of particular interest to plastic surgeons in facelift cases since its size can improve the buccal appearance of the aging face [8].

The main advantage is that the Bichat fat pad has its own structural features as a blood circulatory system and interlobular connective tissue that enable avoidance of its damage during various surgical maneuvers [11]. However, special care should be taken during preparation to spare small blood vessels and gently dissect the fatty mass in order to preserve its function. The capsules overlying the fat pad should not be torn so as to maintain its volume and shape as well as enable its suturing to the bone defect. The arteries and veins overlying the fat pad should be preserved. Some authors advise that the Stensen’s duct should be identified with a lacrimal probe before incision to avoid damaging it during the procedure [13]. Most surgeries are made with a pedicled BFP to ensure its good condition. There are also quite different approaches known, especially those using the BFP as a free, not pedicled, transfer. Resorption of the BFP used as a free graft is limited, as reported by some authors, so over-augmentation is not recommended [12,13,14].

There are few surgical techniques and approaches for the BFP known and used. Fagan et al. mentioned three surgical approaches. The first one, called Matarasso’s method, is the incision of buccal mucosa 1 cm below the opening of the parotid duct. Another one, known as Stuzin’s method, consists of an incision behind the opening of Stenson’s duct. And the last one, the most recommended method, is an incision at the site just superior to the gingivobuccal sulcus [1,6,12,13]. This method is widely known and popular. Intraoral blunt dissection can be made in close relation to the surgical defect, via the same incision, or other maneuvers can be used to ensure the BFP’s easy access point [5,6,7,8,9,10,11,12]. Mannelli et al. harvested the BFP by making an intraoral blunt dissection anterior and medial to the coronoid process while carefully spreading the fascia and the fat pad. Care should be taken to gently and precisely enable the pedicle preparation to the maximum extent of its supporting vascular plexus and stroma. The author used the periosteal tags in advance to suture the most advanced portion of the fat graft. This operation can be performed with one incision, affecting neither the appearance nor the function of the area, which underlines the ease and availability of BFP technique usage [13]. The BFP also can be approached directly through an existing surgical defect. If the defect is located in the mandibular retromolar area, the BFP can be approached by making a vertical incision in the posterior buccal mucosa, lateral to the ascending ramus, and posterior to the Stensen duct orifice [10]. According to Bereczki-Temistocle et al.’s research, patients can be successfully treated with a BFP flap. Single-layer sutures could improve the reduction in the vestibule without any signs of necrosis [15]. Suturing the first layer with mattress sutures improves its good and stable position. The second layer of sutures is mostly consisted of the interrupted sutures once [10,11,12,13,14,15,16].

In summary, some special surgical steps need to be highlighted. First, the most commonly used approach is the intraoral approach, when the BFP is located in the distal part of maxillary tuberosity. A single mucosal incision followed by a periosteum stab incision and scissor blunt dissection is sufficient to expose the BFP. When wide exposure to the fat is achieved after additional blunt scissor dissection, surgical tweezers and mosquito forceps should gently mobilize the BFP in a circular movement. Because of the following, the BFP does not tear and has a good structure. In the case of a double-layer closure, with the BFP and mucoperiosteal flap, a full flap design should be handled with care so as to not tear the periosteum and cause a slight disturbance in blood flow.

## 4. Indications and Contraindications

The Bichat fat pad’s anatomical proximity to the location of various intraoral defects makes it a flap of choice in various congenital and acquired defects that occur in the maxillofacial area. The shape, size, and volume of the BFP might vary individually, the same as its usage for different sizes of surgical defects within its proximity. Before any surgery, a detailed patient examination is necessary. Additional CT/MR imaging might be helpful to identify and measure the size, shape, and location of the BFP and plan the spectrum of each surgery.

A major limitation of the BFP is related to the status of the bone defect or a wound in the oral cavity. The microbiological status of the wound or bone defect is very important. A clean wound, without any signs of irritations, inflammations, and local contamination, can be easy, with a good overall success rate, and closed primarily during one procedure. Microbiological swabs often help in the identification of bacteria, especially those which are atypically found in the oral cavity area. Wounds with pus and microbiological contamination should be firstly threatened pharmacologically and with local debridement. During that time, when a fistula or bone defect is present, a temporary prosthetic rehabilitation could be used. Later on, when the microbiological situation is quite stable, the defect can be closed with a secondary delayed approach. Propper healing in time is not only related to good wound care before and after the procedure. The incision type and wide approach grant not only good visibility but additional blood supply from the mucosal or mucoperiosteal flaps. When sutured, a layer-by-layer suture is advisable. Lack of tension and tension-free sutures support not only the wound’s adequate position at the deeper layer but grant graft immobility [2,3,4,15,16,17,18,19].

There are several listed indications, contraindications, and limitations of buccal fat pad usage. Well-known and often used in clinical practice, BFP flap indication is the closure of oro-antral fistulas. It was originally recommended to use it in cases of small- to moderate-size communications up to 4 cm in diameter [3]. The BFP’s size should enable good closure of the defect, regardless if it will be used with a skin graft, covered by a second layer of tissues (the mucoperiosteal flap) or left sutured in situ in the defect [6,9,13,14,15]. It is especially used in fistulas in the vestibular side of the alveolar process rather than on the palatal side due to location issues, the degree of surrounding bone, maxillary tuberosity prominence, surrounding bone volume, or other factors. The BFP’s use is limited to reconstructions in posterior regions of the oral cavity in adults since the flap can not reach the maxillary midline; however, in some cases, when a very wide exposure is granted, this is achievable. Another situation is in children. Kumar proposes that a buccal fat pad can be utilized as an appropriate pedicled flap for coverage after tumor resection in anterior maxillary defects in infants and children [16]. However, the Bichat Ball technique can be used in patients of all ages, including the elderly age, due to specific lipolysis of the tissue. It can be used even in immunocompromised patients due to its stem cell capacity (SC) [15]. The most frequently mentioned advantage is the rich vascularization of the flap. The buccal fat pad can be used as a pedicled flap or as a free graft. The great advantage of this flap is the ability to be keratinized in time when used for oral cavity reconstruction [14]. The fat cells act as stem cells, and this flap may change into any kind of tissue given the right circumstances [13,14,15,16,17]. When used to close oral wounds, it transforms into healthy oral mucosa. Complete epithelialization of the BFP can be observed within even 1 month [15,17].

The contraindication to BFP use in oroantral fistula closure is in cases when bone reconstruction is necessary to place and plan dental implant rehabilitation, where hard tissue is required [13]. The BFP can be used just once per side; an already previously used buccal fat pad flap is a contraindication. In cases of chronic sinusitis or purulent inflammations following oroantral fistula treatment, the BFP should be used when inflammation is limited. Some authors even advise first maintaining the inflammation process with antibiotic therapy and postponing the final surgical treatment until the infectious process is stabilized [6]. Very slim or skinny persons might have an underdeveloped BFP, causing limitations in their mobilization and usage in larger defects [5,6,7,8,9,10,11,12,13,14,15,16,17,18]. From the author’s perspective, a very wide elevation of mucoperiosteal flaps improves the flap length and its mobilization and decreases vestibule and buccal recess reduction in volume.

Both occurrences of oro-nasal or oro-antral communication can be closed either simultaneously, in primary surgery, or closed in a secondary approach. Late closure has special considerations. This finding might require the incision of a fistula, wound debridement, excision of some tissues, and preparation of a good refreshed tissue surface for its later proper closure. Both newly formed oro-antral/oro-nasal connections and chronic fistulas require an individual approach. Closure of chronic fistulae in radiating areas or scar tissues after past surgeries may be challenging. Tissue scarring, contraction, and a lack of soft tissue material are serious problems. Oral cancer patients represent a big chapter for BFP usage. Previous head and neck radiation treatment does not represent an absolute contraindication to BFP use. A study by Bereczki-Temistocle et al. showed a statistically significant difference between healthy patients and patients with a history of radiotherapy regarding relapses and BFP usage [11,12,13,14,15]. Furthermore, ablative oncological surgeries, followed by radiotherapy (lack of adequate vessels and arteries and tissue condition), might greatly decrease the number of possibilities to treat such situations in oncological patients; however, the usage of the BFP might be a solution in the following cases [12,13,14,15,16,17]. From the authors’ point of view, tissue and wound debridement, followed by adequate sutures layer by layer, grant a very good and stable surgical outcome.

The most common complications described were represented by flap partial necrosis, its perforation; local infection; excessive local scarring, especially in cancer patients undergoing adjuvant radiotherapy treatment; late wound dehiscence; and oroantral or oronasal fistula reoccurrence, where patients’ comorbidities and wrong indications for surgery were the main influencing factors. Other possible complications mentioned in the literature are trismus, limited mouth opening in time, facial swelling, hematoma, abscess formation, and ecchymosis on the buccal area [13,14,15,16,17,18]. Very rarely, damage to the parotid salivary duct or a major artery might occur. However, complications are not common, and the method is popular. Gonzalez et al., in their prospective study, came to the conclusion that patients were highly satisfied overall with the treatment and with phonetics, esthetics, and chewing after BFP usage [19]. Examples of various uses of the Bichat fat pad might include its role in not only maxillary/palatal defects closure but also its usage in the temporomandibular joint, orbital floor, alveolar bone, zygomatic and buccal area, etc. [15,16,17,18,19,20].

Further, atypical limitations include slim patients with underdeveloped or small portions of the BFP. This is not only related to decreased fat volume but also very troublesome BFP preparation when it is small and well attached to its ligaments and surrounding soft tissues. In that situation, even suturing the BFP is troublesome because of its limited mobility and the necessity to use deep-layer sutures. Later increased tension might impact post-operative cheek movements. Second, the BFP tends to tear or cause wound dehiscence with its sutures with tension.

## 5. Oroantral Fistula (OAF)

The oroantral fistula is the name of the pathological connection between the maxillary sinus and oral cavity, lined with the epithelium. This type of connection is present when it is not diagnosed, found, and properly maintained during a procedure in the oral cavity. Oroantral communication (OAC), on the other hand, is a type of communication present in the oral cavity for no more than 24–48 h after surgery, trauma, or other similar events. After that time, the OAC starts to reorganize itself and forms fistula (OAF), which is a common complication after dental extractions in the posterior maxillary bone, mainly in the region of molar teeth. This epithelialization usually occurs when the perforation persists for at least 48–72 h [6,14,15,16,17,18,19,20]. Defects smaller than 2 mm can heal spontaneously via secondary healing with blood clots, whilst larger defects require surgical treatment. The formation of a fistula may be a consequence not only of the extraction of maxillary teeth but also a complication of any other procedures. For example, as listed in Bereczki-Temistocle’s retrospective study, a similar occurrence of OAF/OAC might be present after a cystectomy, poor implant placement, some periodontal procedures, inadequate bone augmentation, and even a sinus lift procedure [15].

Similar fistula or communication can be visible between the oral cavity and the nasal cavity, forming either oro-nasal communication or fistula. Its presence requires more precise surgical planning, quite often combined and improved by two or more local flaps (palatal flaps, palatoplasty advancement flaps, rotation flaps, the BFP, etc.) [15,16,17,18,19,20].

Commonly presented fistulas are closed with flaps which include the buccal or palatal tissues. These methods are not free from limitations. The following fact encourages practitioners to reach for other solutions, especially if previous mucosal or mucoperiosteal flaps were not satisfactory, with inadequate blood supply, and with visible scarring or defect tissues caused by past surgeries. Usage of the most popular buccal flap technique is restricted to conditions when the defect has been dislocated to the palatine area due to a greater vestibular sulcus loss. However, Amaral et al. do not agree that more palatal placed defects are in relation to the maintenance of the furrow depth related to the use of the Bichat fat pad. Researchers observed loss of furrow depth, but to a lesser extent compared to that observed with the techniques of using vestibular flaps alone. The reintervention with the buccal advancement flap may have failed due to the scarred buccal flap’s poor quality and possible inadequate blood supply [6,15].

**Figure 1:**
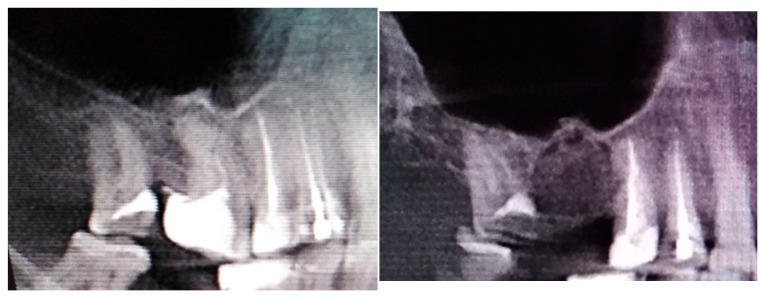
A case after the removal of the first upper right molar and curettage of some periapical inflamed tissue resulting in the loss of buccal cortical plate and opening of a huge oro-antral communication (CBCT scans in sagittal view).

**Figure 2:**
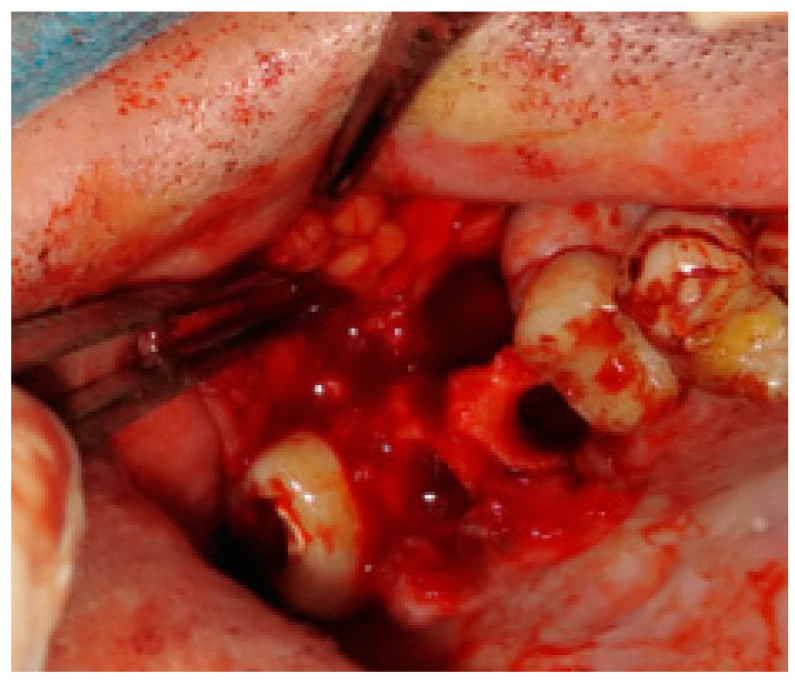
The bone defect with visible buccal cortical bone loss. In this case, when the patient is not willing to undergo any dental implant rehabilitation, it is possible to suture the BFP in a double-layer technique to fully close the bone deficiency and cover it with a sliding muco-periostal flap.

**Figure 3:**
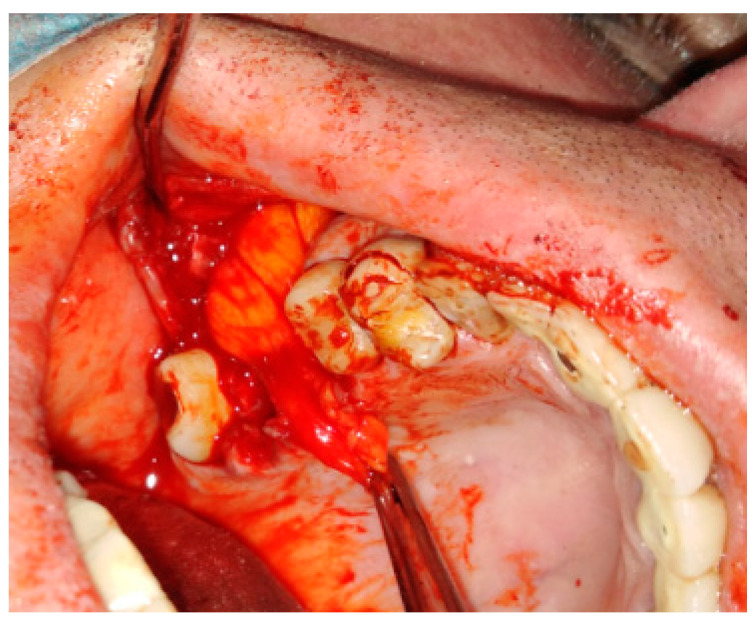
The bone defect was covered with the Bichat fat pad after mobilization. The entire defect will be sutured with vertical mattress sutures to the palate to ensure its proper position.

**Figure 4:**
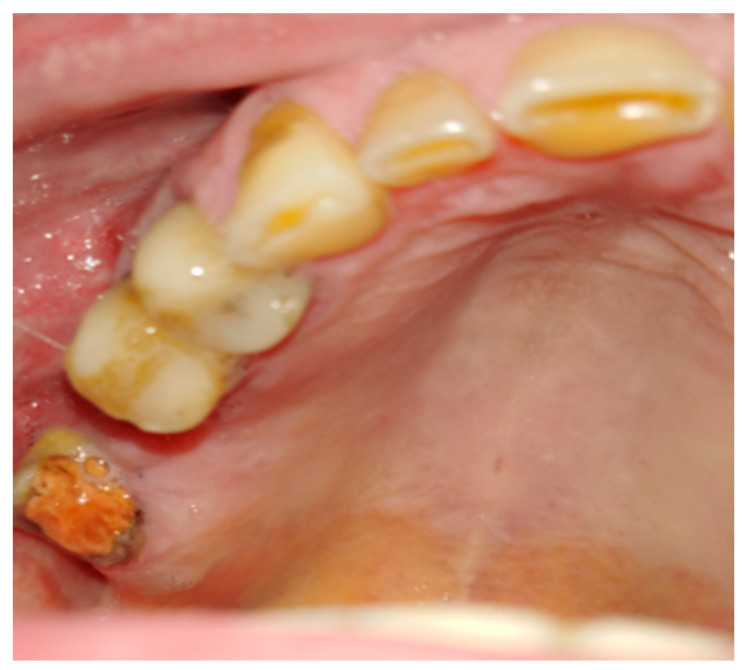
A very good result with limited buccal corridor narrowing and good tissue volume at the top of the dental arch.

The rotation of the palatal flap can be used to solve some problems with the closure without buccal depth loss and esthetic function reduction. A full-thickness flap that contains the palatal artery is a good option to close an oro-antral communication. However, this procedure creates great morbidity and requires a long post-operative care period of the donor site on the palate. Flexibility possibilities of the palatal flap are limited, which forces the surgeon to create a large incision to assure the rotation of a sufficient amount of tissues. Second, the palatal defect heals via granulation, which is also troublesome for the patient. The location on the vestibular sulcus of the maxillary alveolar bone and the large size of the lesion support the choice of the pedicled buccal fat pad flap technique [21]. In some cases of bigger maxillary sinus wall deficiencies, the suturing can be troublesome, especially when it causes severe buccal narrowing or is sutured with tension. A free-of-tension suturing of any oroantral communication is easily achieved with the BFP and can be easily used for suturing in any dental surgery (Figure 1, Figure 2, Figure 3 and Figure 4).

## 6. Maxillary Wall Defects

**Figure 5:**
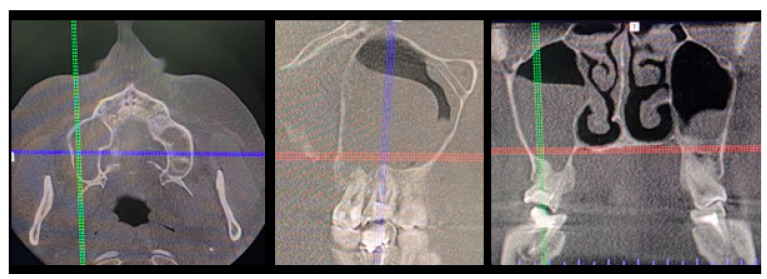
The inappropriate endodontic treatment of the upper second molar led to its inner root resorption, forming of granulation tissue, and the co-incidence of the right maxillary sinus retention cyst. The inflammation and granulation tissues, adjacent teeth, and bone were scheduled for removal. Because of combining wound debridement, cyst, and teeth removal, from the same approach, few different procedures were made. Wound debridement and removal of granulation and inflamed tissues, along with some bone, were scheduled. Because of the presence of oro-antral communication, resulting from the loss of wast bone tissue, the Bihat fat pad was used to close the communication and suture the wound in two layers. The blue, green, and red reference lines are related to CBCT evaluation in axial, sagittal, and coronal views respectively.

**Figure 6:**
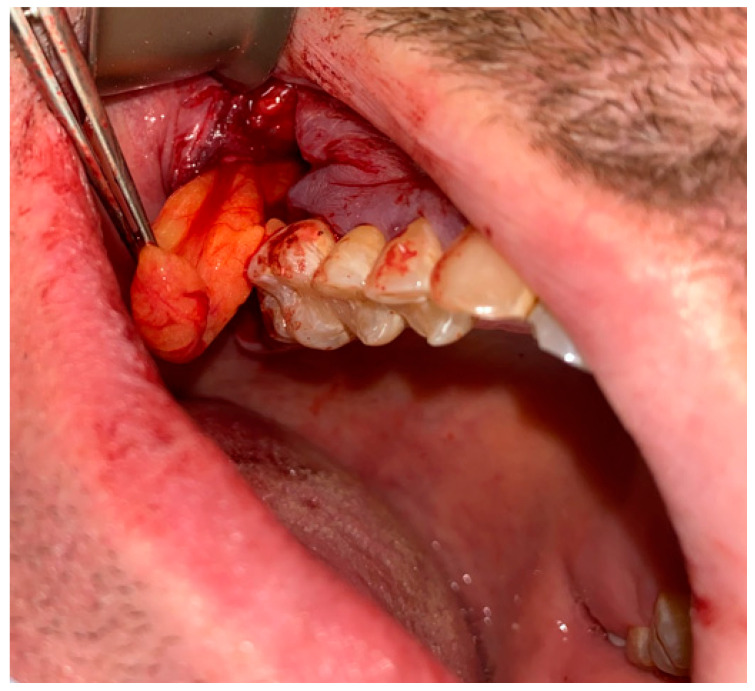
Bichat fat pad mobilization to cover the bone defect and close the oro-antral communication.

**Figure 7:**
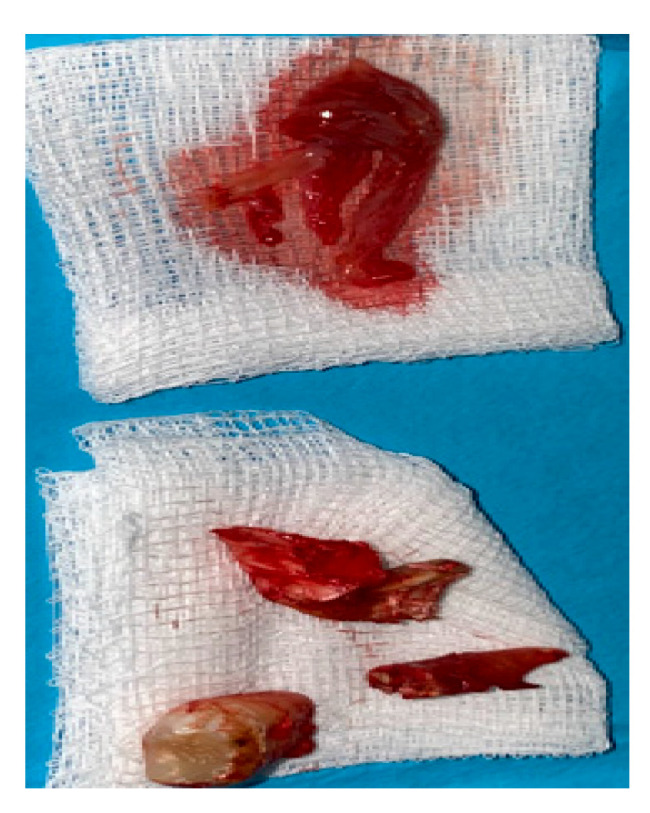
The surgical specimen consisted of granulation tissue, bone with inflammatory changes, and sinus retention cyst.

**Figure 8:**
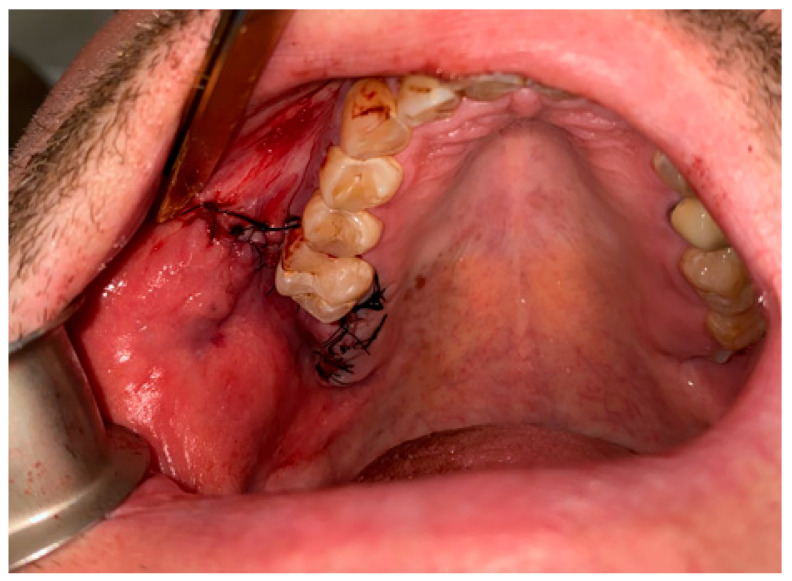
Proper suturing of bone defect and closure of the communication between the oral cavity and right maxillary sinus.

In some special cases, the usage of mucoperiosteal flaps can be insufficient to cover the bone defects. Then, additional usage of the BFP can improve the surgical result (Figure 5, Figure 6, Figure 7 and Figure 8). Various clinical situations can lead to defects in the walls of the paranasal sinuses. Many techniques for defect coverage are known; however, the BFP is considered a very good material for most vertical, horizontal, and combined bone defects in the palate–maxillary area [9,15,16,17,18,19,20,21,22,23,24,25]. The fat pad can be used in different techniques and alterations. Choi et al. presented a case of a patient clinically diagnosed with a dentigerous cyst (DC) associated with a supernumerary tooth in the maxillary sinus. Authors reported their experience with the innovative bilateral pedicled sling BFP flap for intraoral reconstruction after the removal of a larger recurrent DC in the maxillary sinus. The BFP flaps were rotated and covered the alveolar and anterior maxillary defect following cyst enucleation with the Caldwell–Luc procedure [22].

Maxillary wall defects may be a consequence of sequestrectomy in the treatment of medication-related osteonecrosis of the jaws-MRONJ. This situation is considered to be a severe adverse reaction to drugs used for the management of cancer, osteoporosis, or other diseases. MRONJI is exclusively seen in the maxillofacial region, probably due to the high vascularity and rapid bone turnover of the jaws in response to mechanical stress. Surgical treatment consists of complete removal of necrotic bone and wound debridement followed by coverage with local or distant flaps. Denes et al.’s case of a patient during bisphosphonate therapy revealed right maxillary sinusitis and oroantral communication after dental implant insertion. Uneventful post-operative healing without wound dehiscence, infection, and necrosis was observed after BFP defect closure. Jose et al. evaluated the effectiveness of the buccal fat pad in the reconstruction of such surgical defects and reported many successful results after the usage of the BFP with or without mucosal coverage [18,19,20,21,22,23]. This finding confirms that the volume of used BFP not only grants a good amount of surgical tissues but is also vascularized enough to heal in the most difficult surgical conditions.

Also, other types of bone inflammation might result in the necessity of some scope of surgical intervention. Oroantral communication formation may be the result of maxillary osteomyelitis of the jaws. One of the possible maxillary inflammations is associated with dental-related factors and results quite often in the formation of secondary findings within the maxillary sinus, like mucous retention cysts or periapical tooth-related inflammations (Figure 5, Figure 6, Figure 7 and Figure 8). The scope of bone inflammation etiology and occurrence requires each surgeon to treat each case individually. Very rarely, in patients with autosomal dominant osteopetrosis (OP), which is a rare metabolic bone disease characterized by a generalized increase in skeletal mass and following increased susceptibility, osteomyelitis of the jaw bones might also occur. In a case reported by Kulyapina, the osteomyelitis of the maxilla resulted in oroantral fistula formation closed surgically with a Bichat fat pad flap [24]. Co-existing findings in the bone, maxillary sinus, and adjacent tissues should be excised simultaneously to avoid unnecessary scaring or tissue adhesions for secondary surgery.

Iatrogenic permanent oroantral fistula can be a common complication after zygomatic dental implant placement. Recurrent sinusitis is an indication of the removal of a failed zygomatic implant, establishing microbiological flora of the sinus, and after wound debridement and sinus surgery, if necessary, the OAC can be closed with the BFP [25].

The BFP can be used as an independent method of closing defects of the sinus wall. Because of the majority of BFP usage, it should also be considered as an option when reconstructing complicated zygomaticomaxillary defects. Chu et al. presented outcomes of zygomaticomaxillary reconstruction with autologous bone grafts supported by a pedicled buccal fat pad flap. A pedicled BFP flap promotes wound healing and prevents the exposure of bone grafts to the maxillary sinus [26]. Kim proposed using a combination of a pedicled buccal fat pad and a resorbable collagen membrane simultaneously with a bone graft for reparation of large sinus membrane perforations over 10 mm occurring during sinus–bone grafts [27].

## 7. Palatal Defects

**Figure 9:**
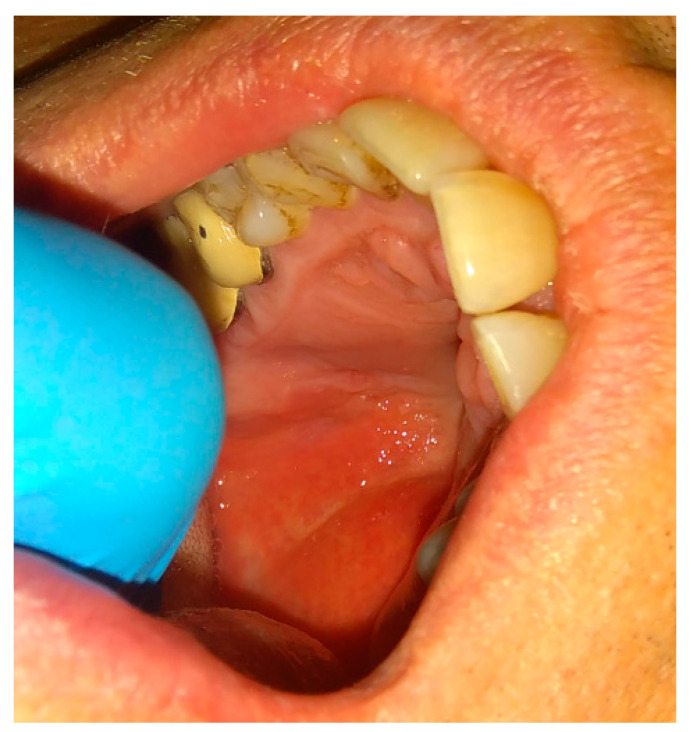
Adeno-carcinoma of the right palate after biopsy.

**Figure 10:**
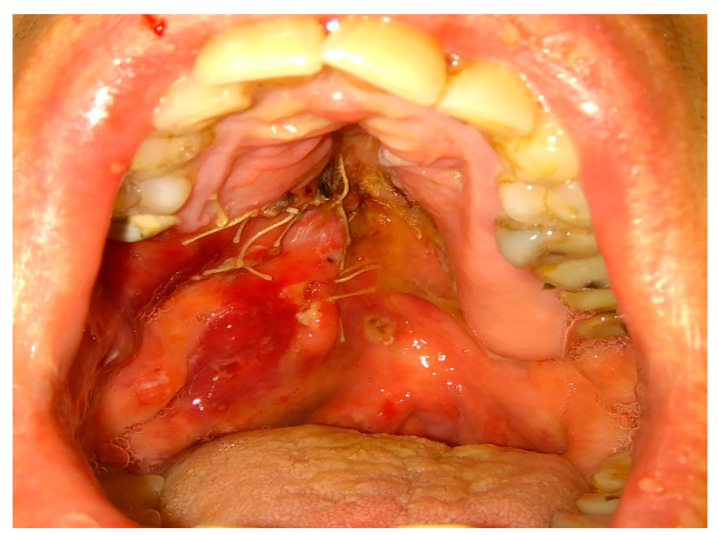
Modified Bichat fat pat and mucosal flaps advanced to cover the defect in the maxillary dental arch, lateral and medial bone wall, and hard palate after resection.

**Figure 11:**
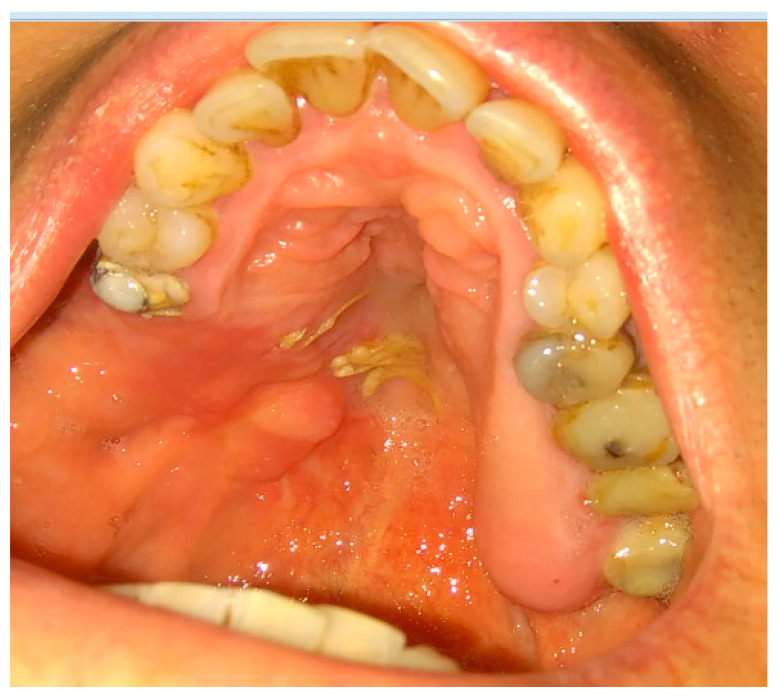
Result after 3 weeks. Notable residual sutures on the palate. A slight discolorization and scarring of BFP after advancement.

A combined mucoperiosteal flap with the BFP can be easily used for suturing not only the maxillary defects but also palatal bone defects in some procedures (Figure 9, Figure 10 and Figure 11). The Bichat fat pad can be useful in both congenital and acquired palatal defects.

Orofacial clefts are common congenital defects of the head and neck region. Adeyemo et al. recommend the BFP method in difficult cases, especially in wide palatal cleft repair, secondary palatal cleft repair, and cases of inadvertent tearing of nasal mucosa during primary cleft palate repair [22,23,24,25,26,27,28]. While it was initially used for fistula closure, its use is generally not only limited to secondary procedures, as mentioned by Saralaya [9,17]. Reports of the use of BPF in cases of the closure of the nasal floor in primary cleft palate repair, oronasal fistula repair following primary repair of cleft palate, and closure of relieving incision defect in primary repair of the cleft palate can be found in the literature [28]. The use of buccal fat flaps for lateral hard palatal defect coverage in primary palatoplasty can reduce the rate of post-operative palatal fistula, especially in cases of the wide palatal cleft. In a case–control study presented by Thanapaisal et al., the buccal fat group showed a significantly lower post-operative oronasal fistula rate and smaller fistula size compared to conventional two-flap palatoplasty [29].

Consorti et al. reported the results of using the submucosal tunneled Bichat fat pad (BFP) flap for primary reconstruction of small–medium-sized surgical defects of the palate after minor salivary glands palatal tumor excision. Complete wound healing after only 4 weeks without complication was observed. The submucosal tunnel for the pedicle passage introduced by the authors reduced discomfort for the patients [30].

## 8. Other Uses

**Figure 12:**
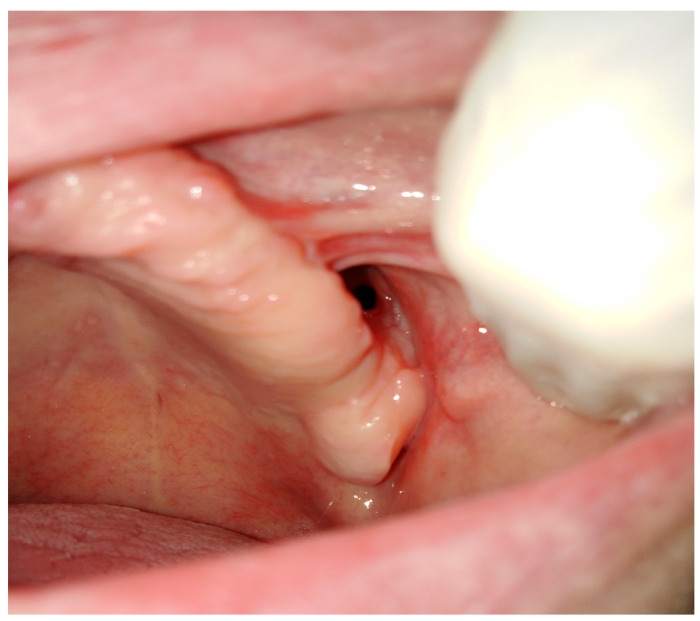
A situation where no methods are good to suture a chronic fistula. Limitations are related to many past surgeries, tissue contraction, scarring, lack of adequate soft tissue, and very narrow area for suturing.

The Bichat fat pad is becoming more popular, not only in maxillofacial surgeries. Esthetic procedures related to the removal of some parts of facial fat grant more esthetic features and a wider facial appearance [26,27,28,29,30]. Despite esthetic usage, the BFP can be used in various surgical procedures. As previously mentioned, the use of buccal fat may be useful for other previously undescribed surgical purposes. It has been used extensively in the reconstruction of oral cavity defects with satisfactory outcomes (Figure 12).

Rattan et al. proposed the use of the BFP as an interposition tissue in the management of temporomandibular joint ankylosis [31]. Temporomandibular joint ankylosis is a pathological condition characterized by abnormal fusion between the mandibular condyle and the glenoid fossa or base of the skull with resultant limitation or complete failure of TMJ movement and mouth-opening ability. As further research proves, a buccal fat pad can be a reliable option for interposition arthroplasty in the management of TMJ ankylosis, and it can be used either as a free graft or as a pedicled flap [32]. Typically, this can also be achieved by the rotation temporalis muscle flap, temporal muscle fascia, or free fat transfer from the abdominal cavity [30,31,32,33].

The BFP began to be of interest to ophthalmologists and surgeons operating in the middle part of the face. Ikekhuamen presented a report on the surgical reconstruction of an orbital floor defect following maxillectomy with a pedicled buccal fat pad [33]. The BFP is not limited to maxillary areas only. In a series presented by Jose et al., two lesions involving the mandibular posterior region were closed by buccal fat tunneling underneath the buccal mucosa to reach the mandible. Although this tunneling is technically difficult, it is equally effective in covering posterior mandibular defects up to 4–5 cm [18].

Bichat fat removal surgery, known as bichectomy, is a procedure used in esthetic medicine to improve one’s facial contour. Some authors recommend extraoral access during facelift surgery. The external approach safely excises buccal fat during facelift dissection while avoiding intraoral incisions and unnecessary contamination [8]. Examples are known to serve not only esthetic but also functional purposes. Bichectomy can be performed to avoid dental trauma to mucosal tissues during the masticatory function, as in the case presented by Montero et al. [30,31,32,33,34].

The BFP’s other usage might include the coverage of small defects in the gingival tissues of posterior teeth and BFP reconstruction for the lateral pharyngeal wall and soft palate in cleft patients and cancer patients or defects from various trauma cases. Nevertheless, larger bone defects from fistulas, clefts, and tumor resection can be closed; however, in some cases, a combination of flaps can be used (like, e.g., the FAMM (facial artery muco-muscular flap) [35,36,37,38,39,40]. The second usage of the BFP is for TMJ reconstruction, orbital augmentation procedures, or even as a filler post-parotidectomy procedure or similar procedures [40,41,42,43,44,45,46,47,48]. The majority of its usage is quite big; however, its usage is mostly related to its size, location to the adjacent tissues, the proximity of SMAS, and its ligaments embedded between various bones and muscles, which might cause some limitations in its preparation.

## 9. Complications and Related Factors

Understanding the potential complications and their management is crucial for clinicians considering the use of the BFP in surgical procedures. Intraoperative complications might include injury to blood vessels or arteries, causing bleeding. Second, the proximity of the SMAS layer and possible damage to facial nerve fibers should be remembered. When performing surgery in the lateral or superior aspect of the parotid gland duct, care should be taken not to damage it. When occurred, its canalization with silicone tube placement for at least 3–4 weeks should be addressed. The issue of the flap having inadequate blood supply or necrosis is not common. Post-operative complications might include swelling, hematoma formation, limited mouth opening, tissue inflammation, and rarely an abscess formation (especially if pterygoid plexus damage, bleeding, and infection occur). The syndrome of a depressed, asymmetrical chick and its slight limitation in movement can sometimes be seen. Rarely, late bleeding appears, especially if the blood accumulates in the pterygoid plexus and maxillary tuberosity area, and nosebleeds when blood accumulates in the maxillary sinus. Palatal wound dehiscence is rare. It can be easily avoided with proper patient education, prescription of a soft diet, and in some special cases, by using an additional prosthetic plate to support the maxillary and palatal bone healing.

## 10. Conclusions

The anatomical location, connective tissue capsule, and morphological structure make the BFP the flap of choice, not only in closing defects in the palatal and maxillary areas. The basis of clinical success is the appropriate qualification of patients for the procedure. The dimensions of the oro-antral communication, general conditions, and associated sinus infection are crucial factors for the success of the surgical treatment. Closure of the oroantral fistula with the BFP is an alternative to the buccal or pedunculated palatal flap, which have limitations. Easy access, rich vascularity, and anatomical proximity favor the use of the BFP in the treatment of bone defects in the maxillofacial region.

## Data Availability

Datasets used and/or analyzed during the current study are available from the corresponding author upon reasonable request.
